# Supplementary material for: Importance of Utilizing Non-Communicable Disease Screening Tools; Ward-Based Community Health Care Workers of South Africa Explain
Source: Int J Environ Res Public Health. 2024 Feb 24;21(3):263. doi: 10.3390/ijerph21030263 (PMC10969901; doi:10.3390/ijerph21030263)
Supplement: Supplementary file 1 [file ijerph-21-00263-s001.zip › ijerph-2779269-supplementary.pdf]

High Blood Pressure Screening Tool

Read the following questions to your clients. Refer them to the clinic for a blood pressure check if the answer to ANY of the questions is YES.

Question	YES	NO
1. Do you have very bad headaches?	Y	N
2. Do you feel tired or confused?	Y	N
3. Do you feel dizzy?	Y	N
4. Do you feel like vomiting?	Y	N
5. Do you have blurry eyesight?	Y	N
6. Do you have chest pains?	Y	N
7. Do you have shortness of breath?	Y	N
8. Does it feel like your heart is beating too fast?	Y	N
9. Is there blood in your urine?	Y	N
10. Do you feel a pounding in the chest, in your neck or ringing in the ears?	Y	N
11. Do you have swollen ankles?	Y	N

Note to CHW: <u>ONLY</u> a clinical health worker can diagnose high blood pressure.

See Health For All Health Promotion Tool pages 134-137 for more on high blood pressure.

Scanned by TapScanner

Warning Signs for Diabetes Checklist

Warning Signs	YES	NO
Do you feel very thirsty?	Y	N
Do you urinate a lot?	Y	N
Do you feel very tired a lot of the time?	Y	N
Do you feel very hungry a lot of the time?	Y	N
Are you losing weight without trying to?	Y	N
Do you have blurry eyesight?	Y	N
Do you have dry, itchy skin?	Y	N
Do you have sores that take a long time to heal?	Y	N
Are you losing feeling or getting a tingling feeling in your feet?	Y	N

Make sure that household members understand the importance of getting help as soon as possible if they have

ANY of these signs, especially if they have any of the risk factors for diabetes.

See Health For All Health Promotion Tool pages 138-143 for more on diabetes.

Scanned by TapScanner

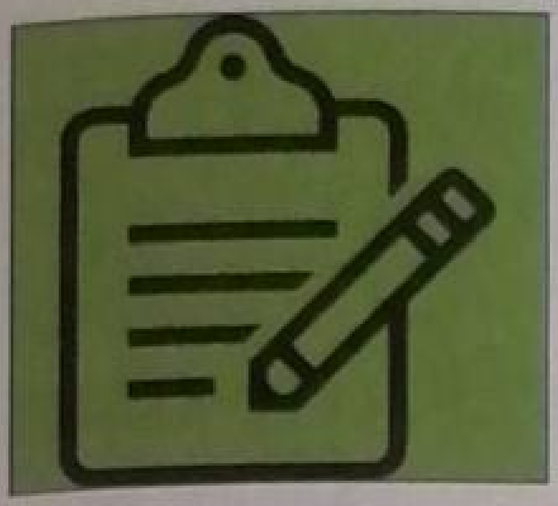

Pregnancy Screening Tool

Read the following questions to your female clients, ages 14-45 or as appropriate. Refer them to the clinic for a pregnancy test if the answer to Question 1 shows that they have missed a period and the answer to ANY of the other questions is YES.

**Question**		
1. When was the first day of your last normal menstrual period? (Your normal period is the period you have every month; this may be different from other women, e.g. the flow may be heavier, the number of days may be different.)	**Day**<br>____	**Month**<br>____
**Question**	**YES**	**NO**
2. Have you been having sex without using any form of contraceptive?	Y	N
3. Are your breasts tender?	Y	N
4. Have you been feeling nauseous?	Y	N
5. Do you feel tired all the time?	Y	N

Note to the CHW: Use the Pregnancy Planner Wheel to determine whether the date of last menstrual period indicates a missed period and possible pregnancy.

Scanned by TapScanner

TB Screening Tool for Adults

Read the following questions to all individuals in the household and refer them to the clinic for TB testing if you tick YES (in the coloured blocks) for any answer.

Question	YES	NO
1. Do you have a cough?	Y	N
If YES, ask: For how long have you been coughing?		
2. Do you have a fever?	Y	N
If YES, ask: For how long have you had a fever?		
3. Have you lost weight?	Y	N
If YES, ask: What do you think might be the cause of you losing weight?		
4. Are you sweating a lot at night?	Y	N

Note to the CHW: Please read the following to the community member.

If you are HIV-positive and you have been coughing for 24 hours, you should go to the clinic as soon as possible for a TB test.

If you are HIV-positive, you should be screened for TB whenever you go for your check-up appointments at the clinic.

See Health For All Health Promotion Tool pages 130-133 for more on TB.

Scanned by TapScanner

TB Screening Tool for Children

Read the following questions to any child and/or their caregiver in the household and refer them to the clinic for TB testing if you tick YES (in the coloured blocks) for any answer.

Question	YES	NO
1. Do you have a cough? / Does the child have a cough that has not improved despite treatment?	Y	N
If YES, ask: For how long have you/has she or he been coughing?		
2. Do you have a fever? /Does the child have a fever	Y	N
If YES, ask: For how long have you/has she or he had a fever?		
3. Have you lost weight? /Has the child lost weight?	Y	N
If YES, ask: What do you think might be the cause of you/she or he losing weight?		
4. Are you feeling tired? / Has the child been feeling tired or been less playful?	Y	N

See Health For All Health Promotion Tool pages 130-133 for more on TB.

Scanned by TapScanner

Treatment Adherence Checklist

	Done	Not Done
**Reviews treatment history, including:**		
• Current regimen		
• Side effects		
• Other treatments		
**Discusses current health status with patient, including:**		
• Overall health and current problems		
• Latest relevant laboratory tests (e.g. CD4 count)		
• Goals for health		
**Assesses patient's medication knowledge, behaviours, and attitudes, including:**		
• Knowledge of HIV/TB/other medications		
• Understanding of drug resistance and implications		
• Attitudes about taking medications		
**Reviews patient's/family's living situation, including:**		
• Daily activities: work, school and travel schedule		
• Eating patterns		
• Access to health centre		
• Special factors: disclosure of diagnosis, medication storage issues		
**Describes proposed medication regimen, including:**		
• Drug names		
• Dosing		
• Food requirements		
• Special instructions/how to give		

Scanned by TapScanner
